# A robust synthesis of reverse Au/ZnO core/shell nanostructures with high visible photocatalytic activity of methylene blue dye†

**DOI:** 10.1039/d5ra03007b

**Published:** 2025-07-21

**Authors:** Nguyen Thi Luyen, Nguyen Xuan Quang, Vuong Thi Kim Oanh, Nguyen Thi Thu Thuy, Tran Quang Huy

**Affiliations:** a Institute of Science and Technology, TNU – University of Sciences Thai Nguyen Vietnam; b University of Transport Technology Hanoi Vietnam; c Vietnam Academy of Science and Technology Hanoi Vietnam; d Phenikaa University Nano Institute, Phenikaa University Hanoi Vietnam huy.tranquang@phenikaa-uni.edu.vn

## Abstract

In this study, we report a robust electrochemical synthesis of reverse Au/ZnO core/shell nanostructures *via* a galvanic replacement strategy, aimed at enhancing the photocatalytic degradation of methylene blue (MB) dye. ZnO nanoparticles were first electrochemically synthesized, followed by the formation of Au (core)/ZnO (shell) nanostructures. The shell formation is attributed to a galvanic interaction between ZnO and Au^3+^ ions, resulting in the reduction of Au and subsequent ZnO encapsulation. The morphology, structure, optical properties, and photocatalytic activity of the resulting nanostructures were systematically characterized. The synthesized particles exhibited core/shell nanostructure with diameters of 30–50 nm, comprising Au cores (25–40 nm) and ZnO shells (5–10 nm). Under visible light irradiation, the Au/ZnO nanostructures achieved up to 98.9% MB degradation within 90 min. The enhanced photocatalytic performance is attributed to the localized surface plasmon resonance (SPR) of the Au core, which promotes efficient photo-induced electron transfer to the ZnO shell and extends light absorption into the visible range.

## Abstract 2

A green electrochemical method enables the synthesis of Au (core)/ZnO (shell) nanostructures with enhanced visible-light-driven photocatalytic activity for dye degradation.

## Graphical abstract

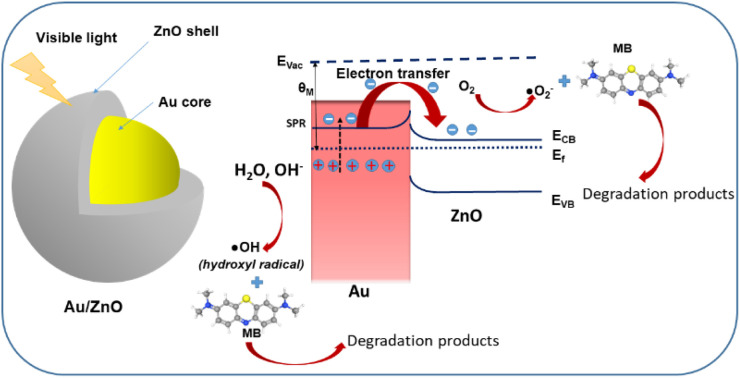

## 1. Introduction

In recent years, metal–semiconductor core/shell structures have attracted significant interest due to their unique optical, electronic and catalytic properties, with potential applications in photocatalysis, photovoltaics, and biosensing.^1–4^ Among various semiconductors, zinc oxide (ZnO) stands out as an effective photocatalyst owing to its wide band gap (3.37 eV), low toxicity, and chemical stability.^5,6^ However, its photocatalytic efficiency is often limited by poor visible-light absorption and rapid electron–hole recombination.^7–9^ To overcome these challenges, ZnO is frequently combined with noble metal nanoparticles such as Au, Ag, or Cu, which serve to enhance light absorption, facilitate charge separation, and prolong the lifetime of photogenerated carriers through surface plasmon resonance (SPR) effects.^10–13^ Noble metal nanoparticles exhibit SPR under light excitation due to collective electron oscillations, resulting in strong localized absorption in the visible region.^14,15^ Decorating nanostructured ZnO with noble metal nanoparticles extends light absorption from the near-UV to visible range. Under UV illumination, the metal nanoparticles act as electron sinks, suppressing charge carrier recombination and enhancing the photocatalytic efficiency of metal–ZnO nanostructures.^16^ Under visible light, noble metals generate SPR-induced electrons that cross the Schottky barrier at the metal–ZnO interface and transfer to the conduction band of ZnO, enhancing charge separation and improving the photocatalytic performance of metal–ZnO nanostructures.^13,16–20^ Various methods have recently been developed for synthesizing Au/ZnO core–shell nanostructures. Notably, Yao *et al.*^20^ employed a two-step liquid-phase pulsed laser ablation technique to prepare ZnO/Au nanocomposites, achieving nearly twice the Rhodamine B photodegradation rate under visible light compared to pure ZnO. Kuriakose *et al.*^16^ used a two-step process combining carbothermal evaporation and sputtering to synthesize Au/ZnO plasmonic nanohybrid thin films, achieving enhanced photocatalytic degradation of 3.7 μM methylene blue within 45 minutes under visible light. In a study, Yao *et al.*^21^ demonstrating high photocatalytic activity toward methyl orange (MO) under UV light.

In contrast to previously reported Au/ZnO nanostructures, which often involve multistep chemical routes or form ZnO (core)/Au (shell) structures, this study introduces a novel and green electrochemical–galvanic synthesis of reverse Au/ZnO core/shell nanostructures. This method uses only zinc and gold bars as raw materials, operating without external chemical reductants (except a very low concentration of trisodium citrate). The unique reverse structure allows the plasmonic Au core to serve as an active electron donor under visible-light excitation, while the ZnO shell facilitates efficient electron transport and pollutant degradation. As a result, the synthesized nanostructures achieve exceptionally high visible-light photocatalytic efficiency (up to 98.9% degradation of methylene blue in 90 min). To the best of our knowledge, this is the first demonstration of such a synthesis strategy and performance enhancement in Au/ZnO core/shell nanostructures, making it a promising platform for scalable photocatalytic applications.

## 2. Materials and methods

### 2.1. Chemicals

Gold bars (99.99%, 50 × 5 × 0.1 mm) and zinc bars (99.99%, 250 × 10 × 5 mm) were purchased from a chemical and jewelry supplier in Hanoi, Vietnam. Trisodium citrate (Na_6_H_5_O_7_) and methylene blue (C_16_H_18_CIN_3_S·*x*H_2_O) were purchased from Sigma-Aldrich. All other chemicals were of analytical grade.

### 2.2. Electrochemical synthesis of ZnO nanoparticles

The synthesis method for ZnO nanoparticles is detailed in a previous publication.^22^ In this process, two zinc bars were cleaned with 3.0% hydrogen peroxide and bi-distilled water, then immersed (20 cm deep) in a 1000 mL beaker of bi-distilled water. To the solution, 0.2 g of trisodium citrate was added and the mixture was magnetically stirred at room temperature. The zinc bars were connected to a DC power supply (LN-ST8008-6K, Lucas-Nülle) as cathode and anode, and electrochemical synthesis was performed at 9 V under stirring for 3 hours, yielding a whitish colloidal solution. The product was separated *via* centrifugation and repeatedly washed with bi-distilled water.

### 2.3. Electrochemical preparation of reverse Au/ZnO core/shell nanostructures

To form the reverse Au/ZnO core/shell nanostructure, two gold bars were cleaned with 3.0% hydrogen peroxide and bi-distilled water. They were then immersed (5 cm deep) in a 60 mL beaker of bi-distilled water, to which 0.06 g of trisodium citrate was added. The solution was magnetically stirred at 100 °C during the electrochemical process at 12 V. ZnO nanoparticle solution was gradually added during the process. The reverse Au/ZnO core/shell nanostructures were synthesized for 5 hours under magnetic stirring at room temperature. The solution color changed from red to purple after the addition of ZnO nanoparticles. The samples were then centrifuged at 6000 rpm and washed three times with bi-distilled water.

### 2.4. Characterization

UV-visible absorption of the aqueous colloidal solution was measured using a UV-vis spectrophotometer (SP-3000 nano, Optima). The morphology of nanoparticles was characterized by transmission electron microscopy (JEM1010, JEOL). The crystalline structure of the nanoparticle samples was analyzed using X-ray diffraction (EQUINOX 5000, Thermo Scientific), energy dispersive X-ray spectroscopy (EDS) was peformed to analyse element compositions in the samples. Surface area was determined by BET (Brunauer–Emmett–Teller) analyses (TriStar II Plus).

### 2.5. Testing for the photocatalytic degradation of methylene blue dye

30 mL of MB solution with concentrations of 10 ppm, 15 ppm, and 20 ppm were prepared in a 250 mL beaker. 5 mL of ZnO NPs or Au/ZnO core/shell nanostructures solution (at concentration of 0.1 mg mL^−1^) was added to the MB solution. The surface adsorption of the substance to MB was examined in dark conditions. After 30 min of dark adsorption, the solution was illuminated with a 200 W incandescent lamp. The photocatalytic method was tested for 120 min. After every 30 min, 1 mL of the reaction mixture was collected and evaluated for the photocatalytic degradation of MB. The reaction mixture was centrifuged at 5000 rpm and the UV-vis absorption spectrum was analyzed to determine the remaining MB concentration. The percentage of MB dye degradation was determined using the following formula:
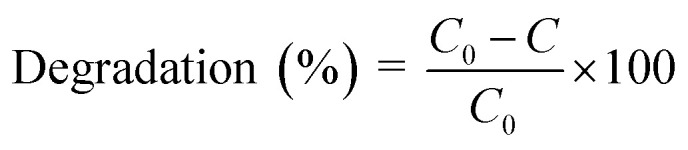
where *C*_0_ is the initial concentration of MB solution and *C* is the concentration of MB solution during the reaction mixture.

## 3. Results and discussion

### 3.1. Optical characteristics

UV-vis absorption spectra were used to investigate particle formation after electrochemistry.

**Fig. 1:**
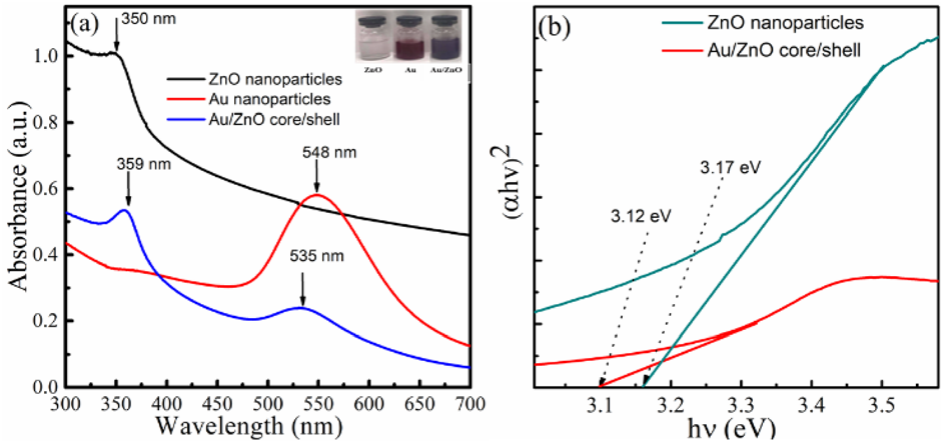
(a) UV-vis absorption spectra of ZnO nanoparticles, gold nanoparticles and Au/ZnO core/shell nanostructures; (b) Tauc plots showing the bandgap energies.

Fig. 1a shows the UV-vis absorption spectra of ZnO nanoparticles, Au nanoparticles and Au/ZnO core/shell nanostructures. The absorption peak for the ZnO nanoparticles was observed at 350 nm, which corresponds to the band-to-band transitions of ZnO nanoparticles. The observed absorption peak and color indicate that ZnO nanoparticles were formed in the solution *via* the electrochemical method. The Au/ZnO core/shell nanostructure sample exhibits broadened absorption peaks at 359 nm and 535 nm. The peak at 359 nm corresponds to the ZnO shell, while the one at 535 nm is attributed to the Au core. The ZnO shell in the core–shell structure exhibits a red shift compared to ZnO nanoparticles, suggesting the formation of a ZnO nucleation layer on the Au nanoparticle surface. The absorption peak at 535 nm, associated with the SPR response of the Au core, shows a blue shift relative to pure Au nanocrystals. This shift is due to the transfer of surface plasmon-induced electrons from Au to the ZnO conduction band, as depicted in the schematic energy structure (Fig. 7). The optical energy bandgap of the prepared samples can be determined using the Tauc relation: (*αhν*)^2^ = *A*(*hν* − *E*_g_), where *α* is the absorption coefficient, *hν* is the energy photon, *A* is a constant related to the localized state tail length, and *E*_g_ is the optical energy bandgap.^13^ The Tauc plot method for a direct allowed transition was applied since ZnO exhibits a direct band gap at the *Γ*-point, as supported by previous optical and crystallographic studies of wurtzite-phase ZnO. Fig. 1b shows the relationship between (*αhν*)^2^ and *hν*, where the *x*-intercept represents the energy bandgap. For ZnO NPs, the bandgap was calculated to be 3.17 eV, while for the Au/ZnO core/shell nanostructure, it was 3.12 eV. This indicates that the addition of Au cores enhances light absorption and gradually shifts the ZnO absorption from the UV to the visible range.

### 3.2. Morphology, structure and surface area

**Fig. 2:**
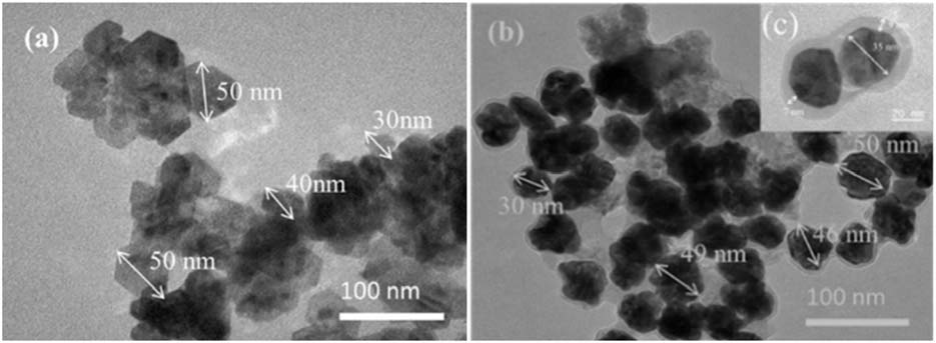
TEM images of (a) ZnO NPs; (b) Au/ZnO core/shell nanostructures, and (c) insert magnified Au/ZnO core/shell nanostructures.

To investigate the morphology of nanostructures, the electrochemical solution was analyzed using transmission electron microscopy (TEM). TEM images of ZnO and Au/ZnO samples are shown in Fig. 2. Fig. 2a shows ZnO nanoparticles sized 25–40 nm formed in the electrochemical solution, while Fig. 2b displays Au/ZnO hybrid particles with a heterostructure, also ranging from 30–50 nm. These particles have a core/shell structure when magnified; the boundary region of the dense particles have an outer shell with a thickness of 5–10 nm (insert Fig. 2c). Based on the electron density, the inner core structure could be Au, and the outer shell could be a ZnO layer. The Au/ZnO core/shell nanostructure can be easily seen in Fig. 1S (ESI).† As a result, the obtained TEM image is completely consistent with the UV-vis absorption spectroscopy. Au/ZnO nanostructures with an Au core and a ZnO outer shell have been successfully formed.

**Fig. 3:**
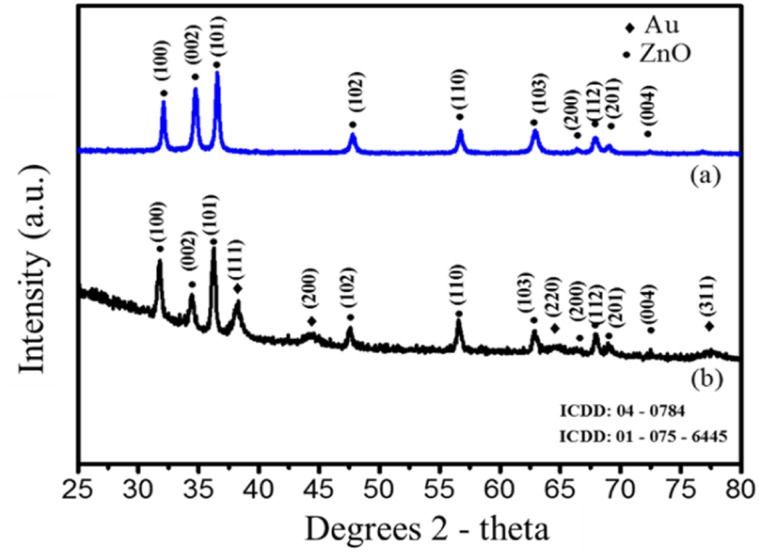
XRD patterns of (a) ZnO NPs and (b) Au/ZnO core/shell nanostructures.

EDS was also performed to investigate elemental compositions of the nanostructures, Fig. 2S† shows the EDX spectra of Au/ZnO core/shell nanostructures. However, to confirm the crystal formation of Au/ZnO core/shell nanostructures in solution, XRD analysis of the structure and crystal phase composition is required. XRD measurements were carried out to support the claim that Au/ZnO core/shell nanostructures were successfully fabricated electrochemically method. The XRD pattern of ZnO NPs and Au/ZnO core/shell nanostructures is shown in Fig. 3. According to ICDD standard card no 01-075-6445, ZnO NPs with peaks at 2*θ* are 31.77°, 34.44°, and 36.26° corresponding Miller index (*hkl*) of (100), (002), and (101) characteristic peaks with hexagonal wurzite structure (Fig. 3a). Other phase peaks do not appear. As a result, the ZnO NPs obtained after electrochemistry are of high purity. Fig. 3b shows an XRD pattern of Au/ZnO core/shell nanostructures, with four additional characteristic peaks of gold with a cubic crystal structure appearing with the indexes (*hkl*) (111), (200), (220), and (311) at 2*θ* = 77.54°, 38.27°, 44.41°, and 64.89°. Both crystalline phases have sharp diffraction peaks, indicating that the material obtained after electrochemical is of high quality and does not exist in an amorphous state. Apart from the characteristic peaks of ZnO and Au, no additional diffraction peaks were observed, confirming the presence of only Au/ZnO nanostructures without impurities. This is consistent with UV-vis and TEM results, indicating that the Au/ZnO core/shell heterostructures are well-crystallized and highly pure in the electrochemical solution.

EDS analysis confirmed the presence of gold, zinc, and oxygen as the primary elements (Fig. 2S, ESI†). While elemental distribution was assessed, the resolution limitations of the instrument prevented clear mapping at the single-particle level. The surface area of photocatalysts are critical parameters influencing light-harvesting efficiency, and accessibility of reactive sites. To assess these properties, nitrogen adsorption–desorption isotherm measurements were performed on the Au/ZnO core/shell nanostructures at 77.35 K. The BET surface area was calculated to be 24.9 m^2^ g^−1^ (Fig. 3S, ESI†). These structural features support the observed high photocatalytic performance and reinforce the functional role of the ZnO shell morphology.

### 3.3. Growth mechanisms of reverse Au/ZnO core/shell nanoparticles

Crystal growth in the aqueous solution occurs in two steps. Step 1: synthesize ZnO nanoparticles. The electrochemical process with the zinc electrode is Zn^2+^ ion mass generated at the anode that dissolves into the solution. Zn^2+^ ion is reduced immediately in solution to form Zn atoms. Because Zn atoms a highly reactive, it is easily oxidized to crystallize in solution to form ZnO NPs. Reaction equations are followed as (1)–(3):

1 Zn − 2e = Zn^2+^

2 Zn^2+^ + 2e = Zn^0^

3 2Zn + O_2_ = 2ZnO

Step 2: synthesize reverse Au/ZnO core/shell nanostructures by electrochemical method from bulk gold electrode based on the galvanic replacement reaction. This step is divided into two sub-processes. The first process takes place at the gold electrode to produce Au^3+^ gold ions that dissolve into the solution according to the eqn (4):

4 Au − 3e = Au^3+^

The second process is when ZnO NPs solution is introduced into gold electrochemical solution to form reverse Au/ZnO core/shell nanostructures upon heating. Under the thermal annealing effect, ZnO NPs dissolved melts *vs.*eqn (5).5
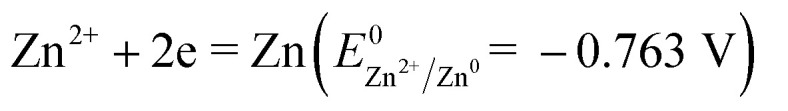


The reduction process takes place according to the eqn (6):6
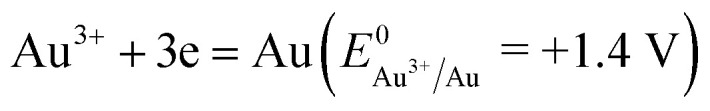


**Fig. 4:**
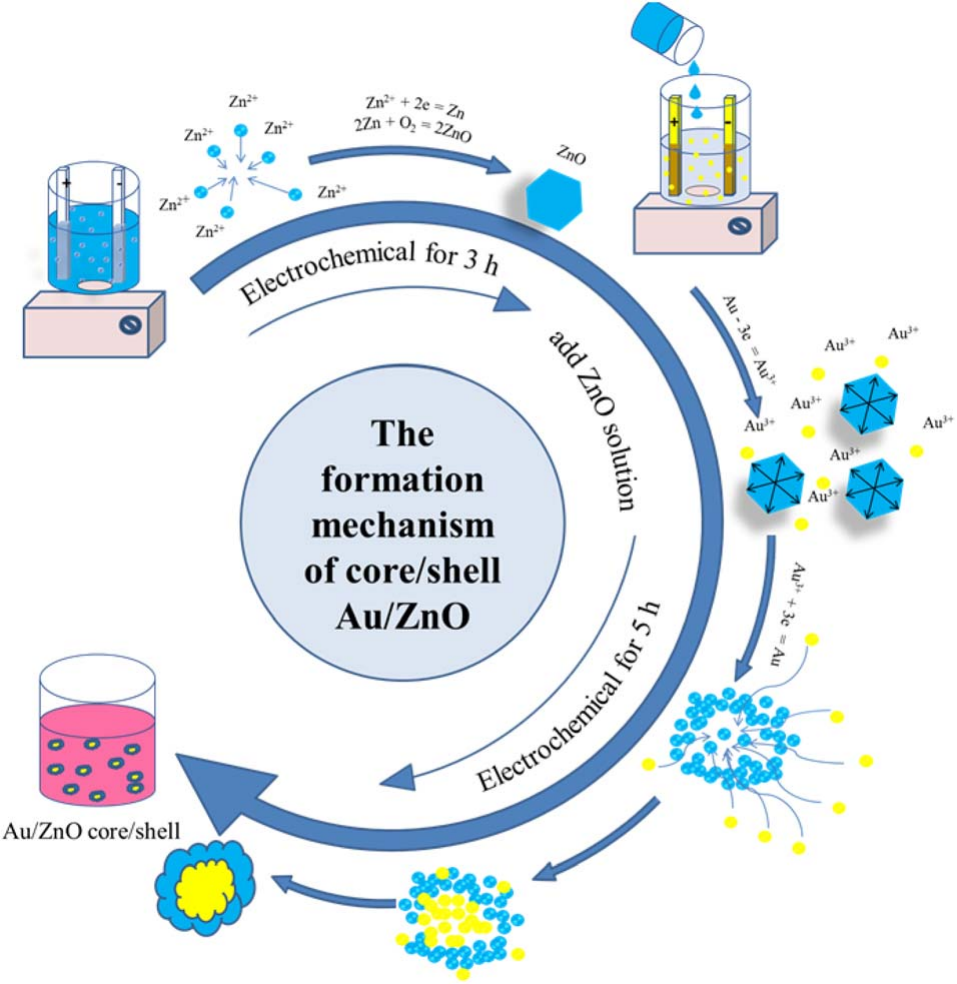
The formation mechanics of reverse Au/ZnO core/shell nanostructures.

The galvanic replacement (electrochemical potential of Zn^2+^ with 
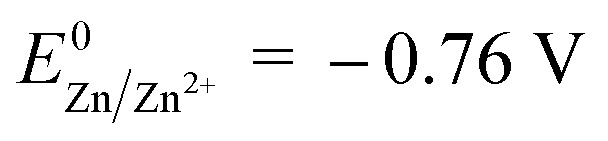
 and 
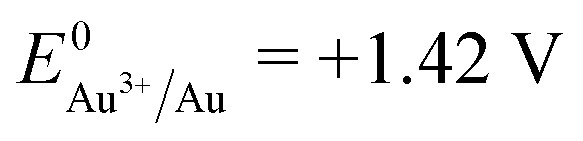
^23^ leads to Au seeds are quickly formed by reducing Au^3+^ ions to Au atoms. Immediately, a ZnO shell is formed around the Au seed to form the reverse Au/ZnO core/shell nanostructures due to an oxidation reaction that involves zinc and oxygen. Fig. 4 illustrates the formation mechanics of reverse Au/ZnO core/shell nanostructures.

The formation of ZnO as the shell rather than the core can be attributed to several thermodynamic and kinetic factors. First, the galvanic replacement reaction is initiated by the oxidation of the Au electrode, generating Au^3+^ ions that are spontaneously reduced by Zn atoms or ZnO surface defects, resulting in Au nanoparticle formation. Once Au cores are established, Zn^2+^ ions in the solution, generated from the decomposition of added ZnO or released during galvanic interactions, undergo re-nucleation and crystallization at the Au surface due to lower interfacial energy, forming a ZnO shell. Additionally, the higher work function of Au and the lower affinity of ZnO for reduction facilitate band bending and Schottky junction formation at the interface, which promotes ZnO coating rather than Au shelling. This structural configuration is further stabilized during thermal annealing and magnetic stirring, encouraging ZnO to diffuse and assemble onto the Au surface.

The formation mechanism of reverse Au/ZnO core/shell nanostructures is driven by the difference in standard electrode potentials, where Au^3+^/Au (+1.42 V) is higher than Zn^2+^/Zn (−0.763 V), enabling a spontaneous galvanic replacement reaction at the Au^3+^/Zn^2+^ interface.

### 3.4. Photocatalytic degradation of methylene blue

**Fig. 5:**
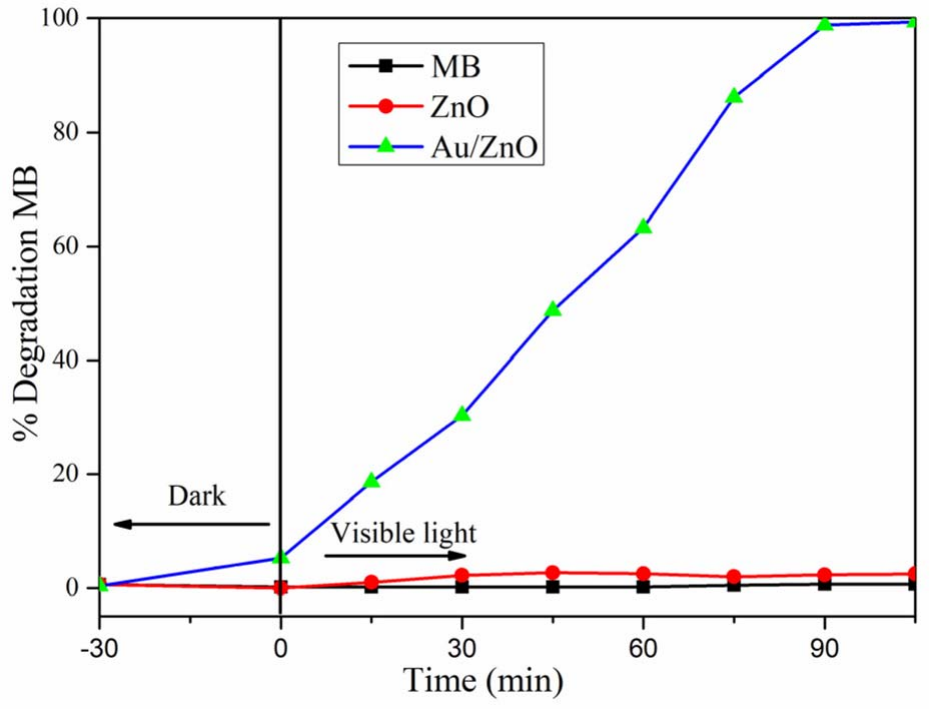
Time-dependent absorbance change of MB in the presence of pure ZnO NPs and reverse Au/ZnO core/shell nanostructures.

The photocatalytic performance of ZnO NPs and reverse Au/ZnO core/shell nanostructures was evaluated by degrading methylene blue (MB) under incandescent light. The percentage of photodegradation of MB with irradiation time is plotted in Fig. 5. The material's surface adsorption to MB was studied for 30 min in the absence of light. The black line depicts the lighted sample with only MB solution (15 ppm), while the red line represents the sample with ZnO added. It is seen that the black and red lines appear to practically overlap and there is no MB decomposition. This is also evidence that using visible light (ℏ*ν* > *E*_g_) irradiated to the semiconductor surface of ZnO NPs, it is not possible to create electron–hole pairs to decompose MB dye. The blue line represents the reverse Au/ZnO core/shell nanostructures added sample, demonstrating that the dark adsorption process of 5% MB was surface adsorbed. When lighted with incandescent bulbs, decomposition efficiency steadily improves. The reverse Au/ZnO core/shell nanostructures completely degrade MB in 90 min. The observed photocatalytic activity is linked to two factors: (1) surface plasmon absorption of Au nanoparticles in the visible region, and (2) electron transfer from Au to ZnO, which occurs because the SPR band position of Au is higher than that of the ZnO conduction band. This facilitates charge separation and reduces electron–hole recombination, thereby boosting catalytic efficiency.^4,24^ As a result, it exhibits a high photocatalytic activity of 98.9%. This demonstrates the enhanced photocatalytic activity due to the presence of the plasmonic Au core, which increases visible-light absorption and promotes electron–hole separation.

**Fig. 6:**
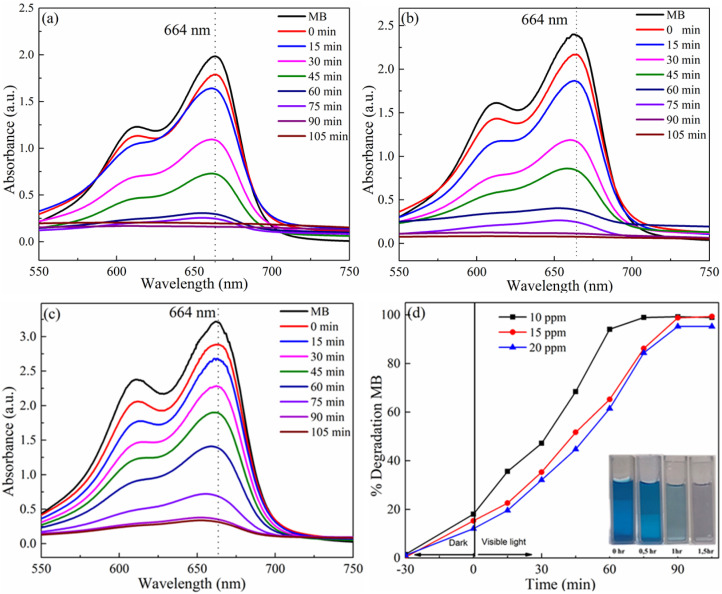
UV-vis absorption spectra of photodegradation of MB dye at concentration: (a) 10 ppm; (b) 15 ppm; (c) 20 ppm; and (d) the % degradation of MB *versus* time using reverse Au/ZnO core/shell nanostructures photocatalyst sample.

Fig. 6a–c presents the variation of absorption spectra of different concentrations of MB solutions in the presence of the photocatalyst of reverse Au/ZnO core/shell nanostructures. From these figures, it could be found that the intensity of the characteristic absorption peak (*λ* = 644 nm) decreased as increased with increasing the incandescent lamp time. The percentage of photodegradation of MB with irradiation time is plotted in Fig. 6d. The black line represents a sample with an MB concentration of 10 ppm, and the decomposition efficiency increases rapidly when lighted. After 75 min, the decomposition was complete. The red and blue lines represent samples with MB concentrations of 15 and 20 ppm, respectively. These two MB concentrations have very comparable photocatalytic degradation rates, and they both degrade fully after 90 min.

### 3.5. Photocatalytic mechanism

The enhanced photocatalytic performance is attributed to the synergistic effects of the Au-core/ZnO-shell architecture. Under visible light, the Au core undergoes surface plasmon resonance (SPR), generating high-energy electrons that are transferred to the conduction band of ZnO, suppressing recombination. The spatial separation of photoinduced electrons and holes across the Au/ZnO interface enhances redox reactions on the photocatalyst surface.

Compared with other reported systems such as Au-decorated ZnO nanorods or ZnO (core)/Au (shell) configurations, our reverse nanostructure delivers superior performance. For example, Kuriakose *et al.*^16^ reported only 62% degradation of MB after 45 min, while our system achieves 98.9% within 90 min. This improvement can be attributed to the optimized band alignment and strong SPR-induced charge injection from the Au core.

To demonstrate the photocatalytic mechanism in detail, it is well-established that both oxidation and reduction reactions contribute to the degradation of MB molecules.^25–30^ The photocatalytic activity of a catalyst is mainly influenced by light absorption, the generation rate of electron–hole pairs, and their separation efficiency. ZnO exhibits strong photocatalytic performance in the UV region due to its effective photo response to UV light.^8,30,31^ However, ZnO performs poorly as a photocatalyst in the visible region, as noted for pristine ZnO. When combined with metals like Au, the metal–semiconductor ZnO exhibits a visible response due to plasmonic photosensitization,^27,29^ enhancing its photocatalytic activity in the visible range.

**Fig. 7:**
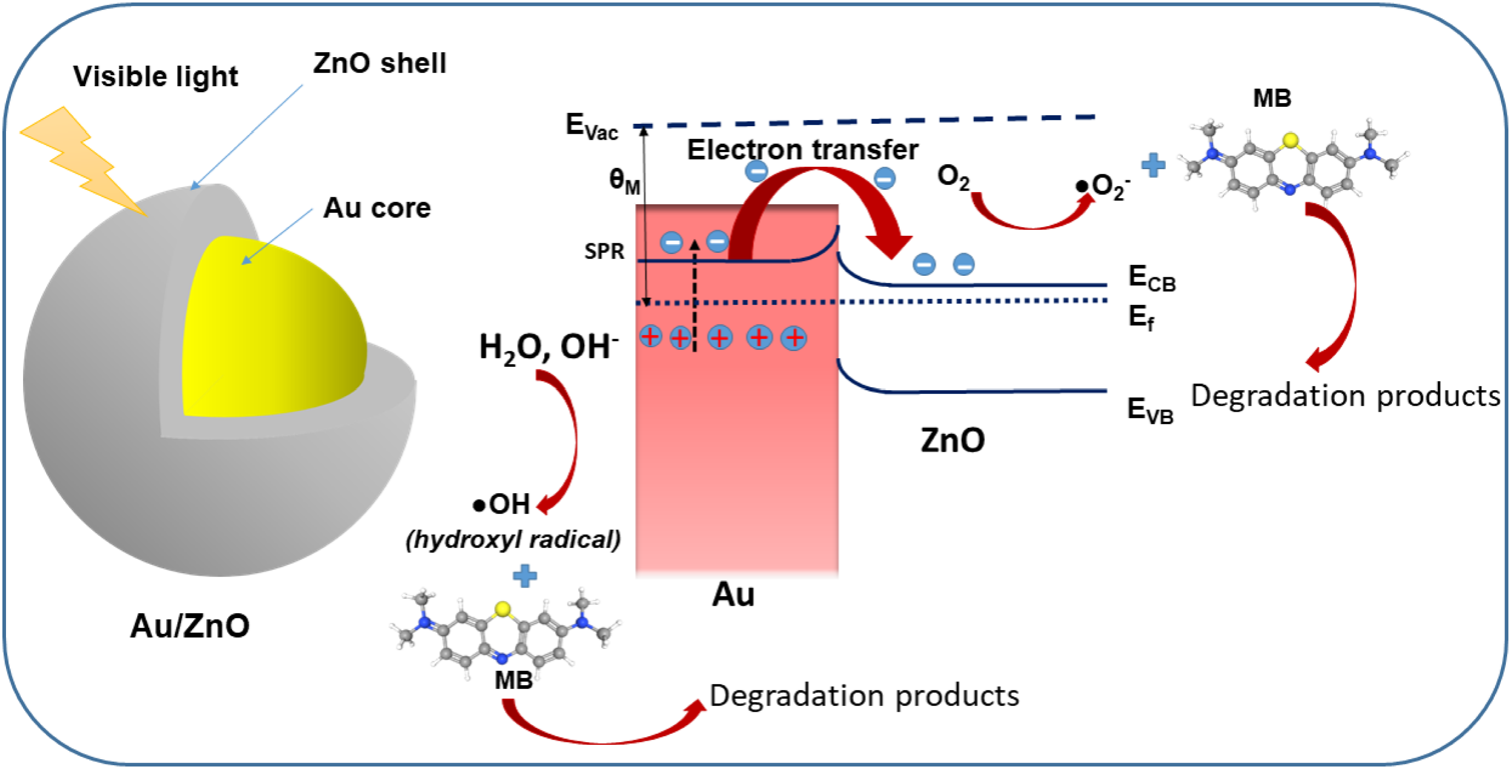
Schematic diagram illustrating energy band alignment of reverse Au/ZnO core/shell nanostructures and proposed processes of generation of charge carriers, migration of the generated carriers and creation of reactive radicals for MB degradation. *E*_f_ = Fermi level, *E*_CB_ = conduction band of ZnO, *E*_VB_ = valence band of ZnO, *E*_vac_ = vacuum level.

The efficient catalytic degradation of MB by reverse Au/ZnO core/shell nanostructures under visible light is illustrated in Fig. 7. The enhanced photocatalytic activity can be explained as follows: the Au core generates SPR-induced electrons under visible illumination. Electrons from the Fermi level of Au are excited to higher states *via* SPR, and some of these electrons overcome the Schottky barrier at the Au/ZnO interface, injecting into the conduction band of ZnO. When Au contacts the ZnO semiconductor, it induces band bending in the energy bands of ZnO, forming a Schottky barrier at the interface. This causes an upward bending of ZnO's energy band. Since the work function (*θ*_M_) of Au exceeds than the electron affinity (*χ*_S_) of ZnO, the barrier height (*θ*_SB_) at the junction is given by the following formula:

7 *θ*_SB_ = *θ*_M_ − *χ*_S_

where *θ*_M_ = 5.1 eV (Au) and *χ*_S_ = 4.2 eV (ZnO the barrier height (*θ*_SB_) is calculated to be 0.9 eV. Consequently, electrons will transfer between Au and ZnO until their Fermi levels equalize.^24,32,33^ The injected electrons in the ZnO conduction band migrate to the surface, where they interact with dissolved oxygen molecules to form superoxide radicals (·O^−^_2_) (eqn (8)). Meanwhile, the electropositivity of the Au core is balanced by electrons donated from the solution, leading to the formation of hydroxyl radicals (·OH) (eqn (9)).

8 e^−^ + O_2_ → ·O^−^_2_

9 h^+^ + OH^−^ → ·OH

These highly reactive (·OH) and superoxide radicals (·O^−^_2_) interact with MB dye molecules, serving as the primary active species for MB degradation. The superoxide radicals (·O^−^_2_) and hydroxyl radicals (·OH) react with MB dye molecules as follows (eqn (10) and (11)):

10 ·O^−^_2_ + MB dye → degradation products

11 ·OH + MB dye → degradation products

Although this study maintains a fixed core–shell dimension, the structure is in principle tunable. The size of the Au core can be controlled by varying electrochemical parameters such as voltage, current density, and reaction time. Similarly, ZnO shell thickness can be modulated by adjusting the ZnO precursor concentration and reaction duration during shell deposition. Such modifications can shift the surface plasmon resonance of Au (typically from 510 to 560 nm), affect the charge transfer distance, and alter the Schottky barrier height at the Au–ZnO interface. These changes would directly impact the optical absorption profile and photocatalytic efficiency. A detailed parametric study on core/shell size effects is underway and will be reported in future work.

## 4. Conclusion

In this work, Au/ZnO core/shell nanostructures were synthesized *via* a green electrochemical–galvanic replacement method without chemical reducing agents. Structural characterization confirmed a well-defined core/shell morphology, with Au cores and crystalline ZnO shells.

UV-visible absorption studies exhibited two peaks: one for the excitonic absorption of ZnO in the UV region and another for the SPR absorption of Au nanoparticles in the visible region. The study demonstrates that the photocatalytic performance of the reverse Au/ZnO core/shell nanostructures reached 98.9% MB degradation after 90 min under visible light irradiation. The photocatalytic mechanism is attributed to the surface plasmon absorption of the Au core in the visible region, enhancing optical absorption and facilitating electron transfer from the Au core to the ZnO shell. These results demonstrate the potential of Au/ZnO core/shell nanostructures as efficient and sustainable photocatalysts for environmental remediation under visible light.

## Author contributions

Nguyen Thi Luyen and Nguyen Xuan Quang designed the experiments and wrote the manuscript. Vuong Thi Kim Oanh and Nguyen Thi Thu Thuy conducted the material testing. Tran Quang Huy contributed to conceptualization, methodology, resources, writing – review and editing, and supervision. All authors reviewed the manuscript, discussed the results, and approved the final version.

## Conflicts of interest

The authors declare no competing interests.

## Supplementary Material

RA-015-D5RA03007B-s001

## Data Availability

The data that supports the findings of this study are available from the corresponding author upon reasonable request.
