# Supplementary material for: A robust synthesis of reverse Au/ZnO core/shell nanostructures with high visible photocatalytic activity of methylene blue dye
Source: RSC Adv. 2025 Jul 21;15(32):25831–8. doi: 10.1039/d5ra03007b (PMC12278265; doi:10.1039/d5ra03007b)
Supplement: RA-015-D5RA03007B-s001 [file RA-015-D5RA03007B-s001.pdf]

Supplementary Information (SI) for RSC Advances.
This journal is © The Royal Society of Chemistry 2025

Supplementary data for

A robust synthesis of reverse Au/ZnO core/shell nanostructures with high visible photocatalytic activity of methylene blue dye

Nguyen Thi Luyen[a§], Nguyen Xuan Quang[b§], Vuong Thi Kim Oanh[c], Nguyen Thi Thu Thuy[d], Tran Quang Huy[d*]

Corresponding author: Tran Quang Huy

Phenikaa University, Hanoi 12116, Vietnam

Emails:

* huy.tranquang@phenikaa-uni.edu.vn (T.Q. Huy)

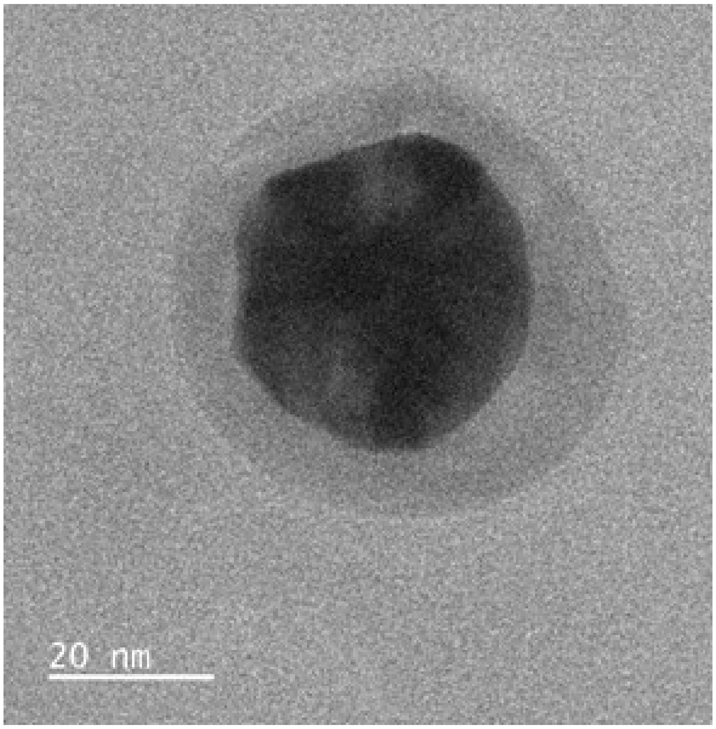

Figure 1S: A typical TEM image of the reverse Au/ZnO core/shell nanostructure

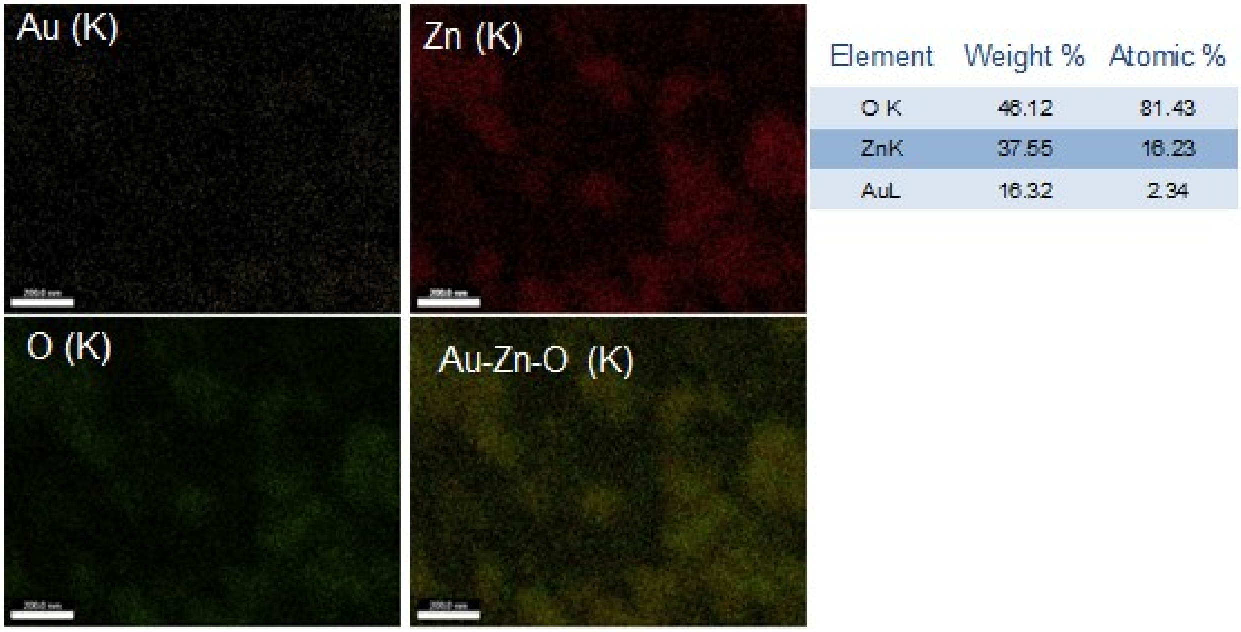

Element	Weight %	Atomic %
O K	46.12	81.43
ZnK	37.55	16.23
AuL	16.32	2.34

Figure 2S: EDS mapping of reverse Au/ZnO core/shell nanostructures

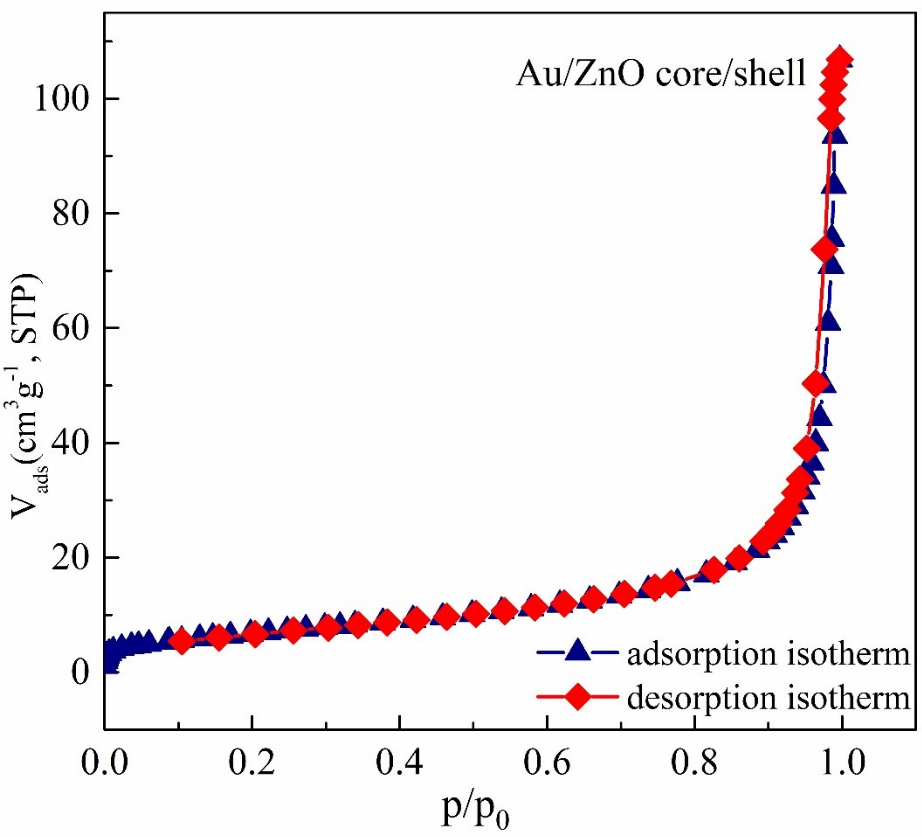

Figure 3S. N2 adsorption-desorption isotherms of Au/ZnO core/shell nanostructures
